# Idiopathic Lymphocele: A Possible Diagnosis for Infraclavicular Masses

**DOI:** 10.1155/2012/593028

**Published:** 2012-09-11

**Authors:** Adenauer Marinho de Oliveira Góes Junior, Salim Abdon Haber Jeha

**Affiliations:** ^1^Division of Vascular Surgery, Air Force Hospital of Belém, 3492 Almirante Barroso Avenue, 66613-710 Belém, PA, Brazil; ^2^Department of Surgery, University Center of Pará (CESUPA), 3775 Almirante Barroso Avenue, 66613-903 Belém, PA, Brazil; ^3^Health Sciences Institute, Federal University of Pará (UFPA), 1 Praça Camilo Salgado, 66050-060 Belém, PA, Brazil

## Abstract

Lymphoceles are usually related to trauma or surgery, and its spontaneous development is rare. The aim of this paper is to report an uncommon case of idiopathic lymphocele located on the right infraclavicular region, in a female patient with no previous local trauma or surgery and unremarkable medical history. The diagnose was suggested by the tomographic aspect of the lesion and confirmed by ultrasound-guided fluid aspiration and cytological analysis. The authors also provide a brief review of the most important thing diagnostic features and treatment options for this condition. For the practicing clinician, the most important is to achieve an accurate diagnose and to choose the proper therapeutic method according to each clinical scenario.

## 1. Introduction

 A lymphocele, also referred to as chylous pseudocysts, cystic lymphangiomas, and lymphatic duct hygromas, is defined as a circumscribed collection of protein-rich lymphatic fluid without an epithelial lining that develops in anatomic compartments as the result of trauma or interruption to the lymphatic system [1–4].

 Lymphocele formation is a described complication of surgery in and around the inguinal and femoral vessels [1], cardiothoracic surgery, blunt chest trauma [1, 2], gynecologic surgery [3, 5, 6], esophagogastrectomy [2], renal transplant [4, 5, 7, 8], and prostatectomy [5, 9] among others. Its spontaneous appearance, on the contrary, is an uncommon fact.

 Currently, no diagnostic algorithm is in place for the evaluation of a suspected lymphocele, but numerous radiographic modalities when used in combination with clinical history and presentation will aid in the diagnose [1]. If imaging modalities provide uncertain diagnose, needle aspiration with cytologic [1, 10], and biochemical [7] analysis may be necessary for confirmation. 

 There is scarce information about the natural history of spontaneous lymphoceles, but it is established that those related to trauma/surgical procedures, when small, are usually asymptomatic and can reabsorb spontaneously [1, 8]; on the other hand, large collections, especially if compressing important structures or when infected, require more aggressive approaches, including fine-needle aspiration, sclerotherapy, catheter drainage, and surgery [1–3, 5]. 

 The aim of this paper is to report a case of idiopathic infraclavicular lymphocele and review the most important diagnosis features and treatment options for this condition.

## 2. Case Presentation 

 Female, 54 years old, without previous noticed diseases, went to the dermatologist complaining about a “node” at the infraclavicular right region. The patient noted the lesions about 2 months before, denied systemic symptoms but referred antiaesthetic complains and local discomfort, caused by a “mass” effect. She also denied previous traumas or surgical procedures nearby the mentioned topography.

 A soft tissue ultrasound revealed a cystic lesion attached to the subclavian vessels; the patient was referred to the vascular surgery team. A more detailed physical exam revealed a visible, well limited mass, painless, firmly fixed at the right infraclavicular region, without fremitus nor murmur, presenting a discreet pulsation (giving the impression of being transmitted from the subclavian artery).

**Figure 1:**
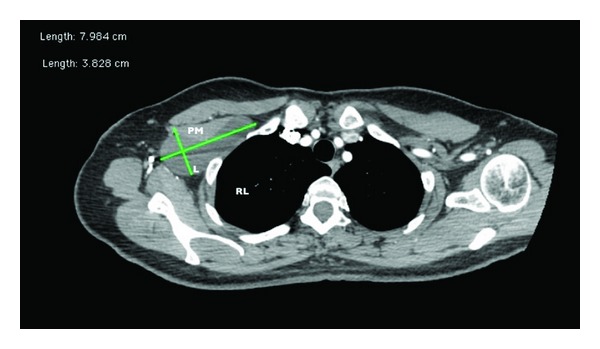
Chest CT with intravascular contrast, axial view. PM: pectoral muscle; L: lymphocele; RL: right lung.

**Figure 2:**
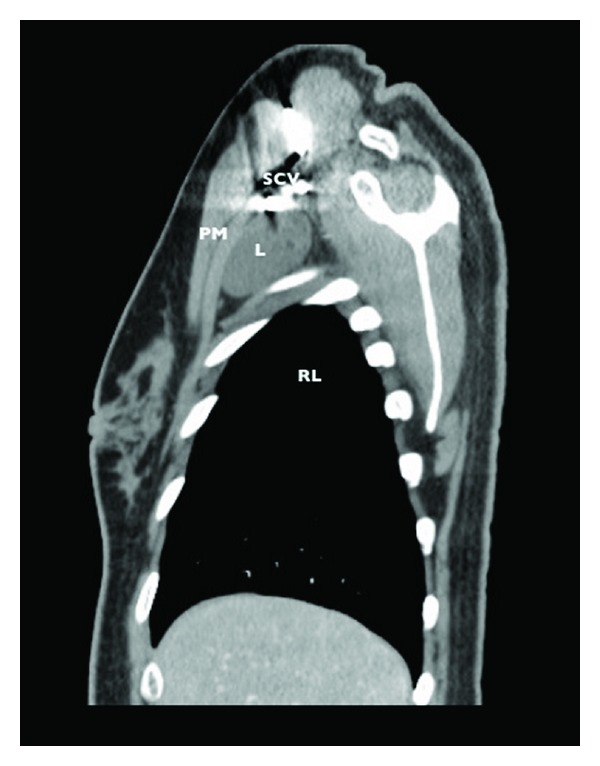
Chest CT with intravascular contrast, sagital view. PM: pectoral muscle; L: lymphocele; RL: right lung; SCV: subclavian vessels.

**Figure 3:**
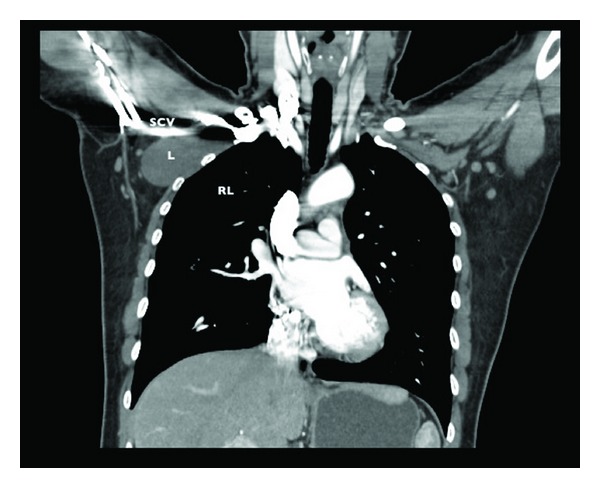
Chest CT with intravascular contrast, coronal view. L: lymphocele; RL: right lung; SCV: subclavian vessels.

 A computerized tomography showed a cystic lesion, under the pectoral muscles, measuring approximately 8 × 3.8 cm, without enhance after intravascular contrast material was administered, on intimate contact to the subclavian vessels trough it's upper border (Figures 1, 2 and 3). The diagnose of lymphocele was suggested by the radiologist physician.

**Figure 4:**
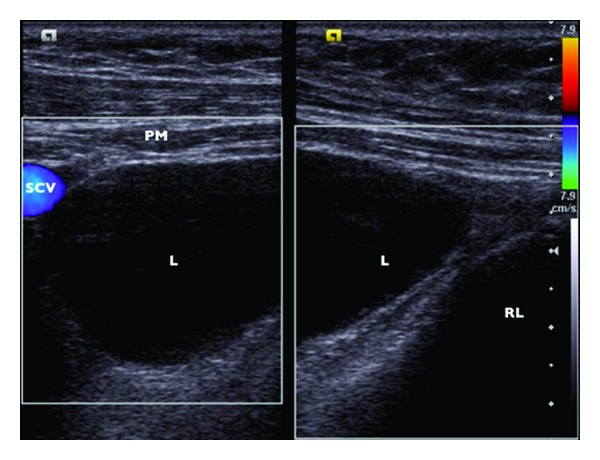
Color doppler ultrasound, axial view. PM: pectoral muscle; L: lymphocele; RL: right lung; SCV: subclavian vessels.

 A fine-needle aspiration, guided by ultrasound was performed. On the application of suction, a clear yellow fluid was obtained. About 20 cc was removed. In spite of the fact that ultrasound examination pointed a remaining volume of approximately 5 cc, because of the proximity to the subclavian vessels and pleura (Figure 4) and the rising risks of iatrogenic damage as the cystic lesion became narrowed by aspiration, the procedure was interrupted.

Cytologic analysis of the fluid showed 100% of normal lymphocytes, confirming the diagnose of lymphocele.

 A post-procedure thorax X-ray was obtained and documented no pneumo/hemothorax.

After the procedure the patient complained no more about the “mass” sensation and discomfort referred before the treatment. 

 After one month the patient remained without complaints and no mass was palpable at the infraclavicular right region. Because of the patient's lack of symptoms, she elected to forgo additional interventional procedures and be followed up clinically.

## 3. Discussion 

 The appearance of idiopathic lymphocele is uncommon and can be only speculated to be associated to lymphatic leakage due to a miss diagnosed minor trauma or repetitive effort injury; its diagnose, evolution, and management are, for this reason, thought to be similar to those that occur after traumatic injuries or surgical procedures. 

Because lymphatic fluid is protein rich and devoid of platelets or clotting factors, a transected lymphatic channel cannot clot and is therefore prone to leakage [1]. A lymphocele may develop days to years after the injury [1]. The natural course of lymphoceles usually depends on its size and the presence of infection [3, 5, 7]. When small, sterile and asymptomatic, the spontaneous resolution by reabsorption is possible [1, 5–8]. However, a few may enlarge and cause symptoms related to infection and compression, depending on the lymphocele location. If intrathoracic, for example, symptoms can include dysphagia, dyspnea or chest pain [1]. When located on the abdomen or pelvis, large lymphoceles may compress the bladder, ureter, rectosigmoid, liac vessels [5, 7], vena cava, portal vein [8], and sacral plexus [9]. The compression of these structures can lead to abdominal distention, abdominal and pelvic pain, hidronefrosis, bladder disfunction, constipation, tenesmus [5, 7] edema in the inguinal regions and genitalia, deterioration of a transplanted kidney, fever, lymphedema of the ipsilateral lower limb, compressive syndrome of the vena cava or the portal vein [8], thrombosis of iliac vessels [5, 7], thrombophlebitis [11], and neuralgia [9]. It is also possible to find severe bilateral peripheral edema and pulmonary thromboembolism from intraluminal thrombus in the compressed inferior vena cava [9, 11]. In the reported case, although the lymphocele had considerable volume and was located near the subclavian vessels and brachial plexus, the patient had no other symptom beside the discomfort caused by the “mass” sensation. 

 Lymphoceles constitute a diagnostic challenge [1], especially when there is no previous history of trauma or surgery, as happened in the reported case. 

 Currently, no diagnostic algorithm is in place for the evaluation of a suspected lymphocele [1]. The diagnosis hypothesis is based on a compatible history, symptoms such as the above described and a variety of radiological exams that help to exclude differential diagnosis such as hematomas, urinomas, seromas, lymphadenopathy, pseudoaneurysms, and abscesses among others. When image techniques are not conclusive or the differential diagnosis with other pathologies is imperative, needle aspiration with biochemical and cytologic analysis is often necessary. 

 Findings on ultrasound include a thin-walled collection [9] with the aspect of a hypoechoic or anechoic well-circumscribed oval structure [3]; occasional internal septa and debris can be also visualized [1].

 The typical presentation on a chest radiograph includes a smooth focal mediastinal mass or mediastinal widening. These abnormalities typically prompt further evaluation with a chest computerized tomography (CT) scan [1].

 CT scan features classically described for lymphoceles include a smooth, oval, or tubular mass with a thin wall that does not enhance after intravascular contrast material is administered, homogeneous attenuation, typically in the range of water and no infiltration of adjacent structures [1–3, 9, 12]. It is important to note that even though the attenuation is usually similar to water, this may alter depending on the chylomicron content [2] and that cysts containing nonserious fluid can have high attenuation on a CT scan and may be mistaken for solid lesions [1]. Calcification of the lymphocele wall may be seen on rare occasions [4, 13].

 Magnetic resonance imaging (MRI) features depend on the fluid chemical composition [2]. Lymphoceles contain proteinaceous fluid similar to extracellular fluid that results in a low T1-weighted intensity greater than water and a high T2-weighted intensity less than water. As the chylomicron content increases, however, there is a reversal of MRI findings with a high T1-weighted intensity and an intermediate T2-weighted intensity [1, 2]. MRI typical findings include thin-walled cystic collections, without contrast enhancement [9], nor evidence of flow, hemorrhage, or hemosiderin deposition [12].

 Although some authors find MRI useful [2], others consider that, for the practicing clinician, the MRI does not provide any additional information to that obtained on the CT scan [1].

 In obscure cases, additional investigations may be found helpful, and lymphangiography and lymphoscintigraphy can be considered [13]. Lymphoscintigraphy allows a two-dimensional visualization of the lymphatic network and is specially useful for evaluation of traumatic lymphoceles [1, 12]. Lymphography can be performed by contrast injection through needle or angiographic sheaths to evaluate the size of the lesion and the presence of internal septation. When found, septum can be broken using guide wires and fluoroscopic guidance with the intention to facilitate the aspiration or drainage of a greater volume of fluid [5]. 

 If the radiographic findings do not correlate or the patient is symptomatic, however, it may be necessary to perform needle aspiration of the fluid and cytologic analysis [1]. Fluid is typically described as straw colored [1, 13], milky color [10], clear and yellow in noninfected lesions and turbid, and gray in infected ones [5]. It contains erythrocytes, lymphocytes, and scant polymorphs [1, 2]. Among the white blood cells (WBCs) there is a predominance of lymphocytes [5] and a 70% lymphocytes of all WBC is characteristic of lymph [10]. Analysis of the fluid demonstrates high triglyceride levels [1], chylomicrons [10], and the same level of proteins, urea nitrogen, creatinine, electrolytes, and lipids as serum has [5, 7].

 Management varies with size, symptoms, and anatomical location. Options include observation, needle aspiration, surgical resection, internal drainage via open or laparoscopic/thoracoscopic marsupialization, and percutaneous external drainage with or without the addition of sclerosing agents [1, 3, 5, 7, 13]. For infected lymphoceles, antibiotics alone are often sufficient [13]. 

 Simple aspiration, preferentially under CT or ultrasound guidance, has been used successfully to treat lymphoceles and relieve pressure symptoms [2, 3, 13]. Needle aspiration has been reported as a safe and effective therapeutic alternative to surgery, and because of its minor morbidity some authors consider it as first-line treatment [13], but most studies report high recurrence rate of 80%–90%, which necessitates repeated aspirations and results in a 25–50% infection rate [5, 7, 13]. In consequence, the use of needle aspiration once only would seem to be sensible [13]. Simple aspiration was first chosen in this case because the patient had an unremarkable medical history and no previous trauma or local surgery and the diagnose of a lymphocele was based only on the radiological aspect. Fluid aspiration, ergo, provided at the same time an opportunity to exclude differential diagnoses and relief the patient's symptoms. 

 Percutaneous catheter drainage has a mean duration of 14.5 days [5]; success rates of 79% to 82% with prolonged drainage have been reported [7], and for some authors success can be as high as 100% [5] even though a 63.6% rate of lymph reaccumulation has also been described [8]. Instillation of sclerosing agents through drainage catheter is an attractive alternative because of its lower reported recurrence rates and shorter duration of therapy than percutaneous drainage alone and because it is less invasive than surgery [11]. Tetracycline, ampicilin, povidone iodine, ethanol, doxycycline, and bleomycin [1, 5, 11] are among the reported sclerosing agents. Sclerotherapy usually results in a success rate of 79%–100% with duration from 9 to 36 days [5]. 

 Surgery has been considered the treatment of choice because of the rates of recurrence after percutaneous aspiration or drainage [11]. Surgical drainage achieves success in 50%–70% and peritoneal marsupialization is effective in more than 90%; however, these methods have some disadvantages, such as surgical morbidity and mortality, economic burden, and the need for long hospitalization [5].

 Idiopathic lymphoceles are infrequently. If even diagnostic algorithm is not currently established, the same is applied for the therapeutic strategies. For the practicing clinician, the most important is the suspicion to achieve an accurate diagnose and to choose the proper therapeutic method according to each clinical scenario.
